# Epigenetic Factors and Mitochondrial Biology in Yeast: A New Paradigm for the Study of Cancer Metabolism?

**DOI:** 10.3389/fphar.2018.01349

**Published:** 2018-11-21

**Authors:** Antonella Stoppacciaro, Serena Di Vito, Patrizia Filetici

**Affiliations:** ^1^Surgical Pathology Units, Department of Clinical and Molecular Medicine, Ospedale Sant’Andrea, Sapienza University of Rome, Rome, Italy; ^2^Institute of Molecular Biology and Pathology, CNR, Sapienza University of Rome, Rome, Italy

**Keywords:** yeast, epigenetic, mitochondria, cancer, reprogramming

## Abstract

Bidirectional cross-talk between nuclear and mitochondrial DNA is fundamental for cell homeostasis. Epigenetic mechanisms regulate the inter-organelle communication between nucleus and mitochondria. Recent research highlights not only the retrograde activation of nuclear gene transcription in case of mitochondria dysfunction, but also the role of post-translational modifications of mitochondrial proteins in respiratory metabolism. Here we discuss some aspects and novel findings in *Saccharomyces cerevisiae*. In yeast, KAT-Gcn5 and DUB-Ubp8 have a role in respiration and are localized, as single proteins, into mitochondria. These findings, beside the canonical and widely known nuclear activity of SAGA complex in chromatin regulation, provide novel clues on promising aspects linking evolutionary conserved epigenetic factors to the re-programmed metabolism of cancer cells.

## Introduction

The recent interest in non-disruptive therapy as a novel approach for treating refractory and metastatic cancers is a new perspective to tackle advanced neoplasia. Rethinking novel therapeutic tools for inducing cell reprogramming and efforts to reactivate cell communications with peritumoral tissues are approaches aiming to reset and reprogram neoplastic cells toward a new differentiation with the control of proliferation, cell to cell and cell to extracellular matrix (ECM) interactions. Although, the identification of single oncogenes and tumor targets boost advances in personalized therapies often tumor recurrence and decreased efficacy of chemotherapy due to high genetic heterogeneity especially at metastatic sites (Gerlinger et al., 2012; Almendro et al., 2014). For these reasons, the pathway-epitope directed approaches is often correlated with the long-term failure of disruptive medicine. The challenge in treatment of non-curable cancer types is therefore to understand complex network of regulatory pathways in term of tissue and organ specific cell communications with the intent to modulate regulatory circuitries. Redirecting cells to differentiation aims therefore to gain a novel apoptotic control avoiding adverse events and toxicity of more aggressive therapies. This principle has been comprehensively defined in the term Anakoinosis proposed by A. Reichle and colleagues (Hart et al., 2015). Here we would like to discuss some clues and novel evidences regarding the epigenetic regulation of respiratory functions found in yeast *Saccharomyces cerevisiae*. Based on the high evolutionary conservation from yeast to man, we can take advantages from discoveries in yeast to find similarities with human cells. Cancer cells reprogram their metabolism to promote growth, survival and proliferation with aerobic glycolysis and show alterations in the mitochondrial functions affecting tumor survival. They also produce macromolecules sustaining enhanced proliferation and ATP is produced in mitochondria thus upregulating glycolysis (Haq et al., 2013; Vazquez et al., 2013). Efforts aiming to understand the ontology of this phenomenon and the causes of the biochemical reprogramming of malignant cells are therefore extremely useful and boost the investigation on new therapy targeting mitochondria. Novel agents able to reverse the Warburg phenomenon or aim at inducing apoptosis via targeting mitochondrial proteins and membranes may offer an innovative therapeutic strategy (Ubah and Wallace, 2014). Metformin, a drug targeting glycolysis, is able to lower the probability to develop cancer as described in epidemiological studies and further assessed in clinical trials. Mitochondria modulate nuclear and mitochondrial gene expression, protein translation and turn over and post-translational modifications (PTMs) play a central role in this concerted regulation. Lysine acetylation seems to be the prevalent PTM of this organelle (Hosp et al., 2017), central metabolic enzymes are acetylated and their dysregulation has a direct impact on mitochondrial functions with implication in human disease and cancer (Lombard et al., 2015). Solid tumors are often poorly perfused and cancer cells are able to survive at very low oxygen concentration (0.5%). Drugs able to block mitochondria and ATP production might represent promising anticancer compounds. Consequently, the exploitation and comprehension of complex molecular processes in mitochondria may become an essential piece of knowledge for an efficient long-term therapeutic approach against resistant tumors.

## Yeast Model and SAGA Complex

**FIGURE 1:**
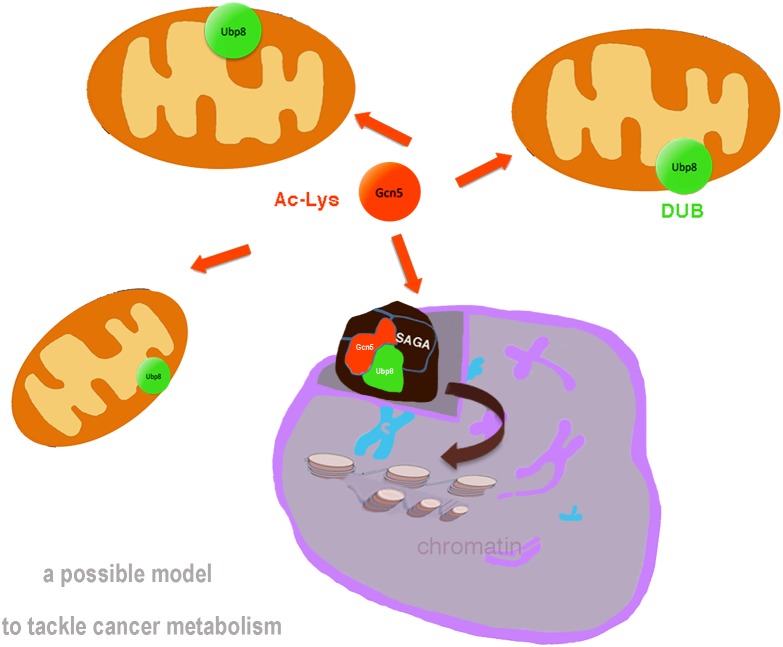
Schematic representation of the canonical role of assembled SAGA complex in the nucleus with KAT-Gcn5 and DUB-Ubp8 modules regulating chromatin structure and gene transcription. In addition, based on novel findings in yeast, we propose a novel role of MT-Gcn5 (orange arrow) and MT-Ubp8 (green) necessary for cell respiration and mitochondrial functions. Collectively, these findings reveal dual functions of these epigenetic factors, as subunits of SAGA complex in the nucleus and single proteins into mitochondria.

In Evolution, proteins conserved to humans represent factors involved in crucial functions of the cell, showing remarkable similarity in structure and function. Great advances may beneficiate of simple model organisms enabling to dissect the complex network of genes and protein interactomes. To this end, yeast *S. cerevisiae* represents an awesome biological model for dissection of complex networks (Cazzanelli et al., 2018) with a variety of molecular and genetic tools. The analysis of genetic interactions and the availability of yeast mutants in genes homolog to oncogenes and oncosuppressors may open to the identification of pathway-level interactions involved in oncogenic risk. Examination of different breast cancer cohorts suggested, for example, that genetic interactions indeed play a role in determining breast cancer risk (Wang et al., 2017). Accordingly, the yeast interactome allows the analysis of regulatory circuitries without the use of animal models. *S.cerevisiae* offers also a comprehensive genome database, SGD^1^ and wide-angle informations such as gene ontology, interactors, phenotypes, expression, and literature for each single gene/protein. Yeast has conserved the majority of fundamental pathways and proteins through evolution and switch fermentative to respiratory metabolism simply by adding glucose or glycerol to the culture medium. In past, *S.cerevisiae* allowed the study of fundamental epigenetic regulators such as K-acetylatrasferase (KAT) Gcn5, deacetylases HDACs, chromatin remodeling complexes etc., The SAGA complex is a multisubunits machinery composed of KAT and deubiquitylase (DUB) modules with subunits involved in the activation of transcription (Koutelou et al., 2010), it is highly conserved and human SAGA/STAGA complex is involved in processes closely implicated in cancer. At transcriptional level, SAGA exerts its widely known activity in the nucleus regulating subset of genes in response to chromatin opening induced by nucleosome acetylation over regulatory regions thus affecting the expression of oncogenes and onco-suppressors in tumors (Wang and Dent, 2014). Mitochondria are also involved in an inter-organelle communication with the nucleus in the so called Retrograde response, that activates expression of genes involved in the switch from aerobic to anaerobic respiratory (Hill and Van Remmen, 2014). The retrograde factor Rtg2 is a component of the SAGA/SLIK complex (Pray-Grant et al., 2002) and promotes nuclear translocation of cytoplasmic factors. The recent discoveries in budding yeast that KAT-Gcn5 (Canzonetta et al., 2016) and DUB-Ubp8 (Leo et al., 2018) are required for respiration and their localization into mitochondria uncover interesting novel aspects of an interplay between single epigenetic factors, not complexed with multisubunits complexes and mitochondria (Figure 1). The need for Gcn5 in respiration in yeast is not surprising considering the importance of acetylation as major PTM of mitochondrial proteins. Absence of Gcn5 or chemical inhibition with KAT inhibitors of yeast cells inhibit the respiratory metabolism in the cell. Accordingly, the human homolog GCN5L1 has been shown to play key role in mitochondrial protein acetylation and modulation of cell metabolism (Scott et al., 2018). Ubp8 is also necessary for yeast respiratory metabolism, is localized into mitochondria as single protein (Leo et al., 2018) and its expression is affected by KAT-Gcn5 and acetylation. Similarly, USP22, hortholog of the Ub-protease Ubp8, subunit of the DUB module of SAGA complex, is overexpressed in liver, breast, gastric, bladder and lung cancers and is a predictor of tumor recurrence in hepatocarcinoma (Tang et al., 2015; Li et al., 2017). USP22 is also considered a marker of metastasis, it induces autophagy and promotes the survival of cancer cells especially in pancreatic cancer (Liu et al., 2011; Yang et al., 2011; Zhang et al., 2011; Ning et al., 2012) enhancing cell proliferation and resistance to chemotherapy under special conditions such as nutrient deprivation or hypoxia. USP22 activity is also regulated by acetylation and other PTMs such as phosphorylation and is a direct target of miR-29 (Huang et al., 2018). Interestingly, it was demonstrated that silencing USP22 inhibited proliferation in bladder cancer cells (Lv et al., 2011) leading to cell cycle arrest, inhibition of autophagy and decreased tumor progression representing a potential target for cancer therapy.

## Epidrugs

Acetylation and Ub-related pathways are therefore involved in regulating cell respiration thus suggesting that PTM inhibitors might been used to target mitochondria. The use of epidrugs targeting K-acetyltransferase and/or ubiquitin related enzymes may represent a novel strategy to affect gene expression through inhibition of SAGA complex and its nuclear, well known role in chromatin remodeling and transcriptional activation. In addition, tumor development is highly correlated with respiratory metabolism and mitochondrial signals influence cell physiology and tumorigenesis. Accordingly, mutations in mitochondrial enzymes can cause the production of metabolites that stimulate cell proliferation (Vyas et al., 2016). It has also been shown that defects in mitophagy are linked to human tumors such as the deletion of Parkin. Targeting mitophagy may therefore offer novel opportunities to inhibit tumor progression (Chourasia et al., 2015). Based on experimental evidences showing beneficial effects related to knockout of USP22 on tumor progression and to inhibition of acetylation we might envisage treatments with KAT and Ub-related inhibitors as active compounds counteracting tumor progression (Nebbioso et al., 2018). Drugs targeting mitochondrial membranes or inhibiting their active states might represent a novel strategy whose efficacy might be combined with directed immunotherapy and/or low dose chemotherapy. This hypothesis is in agreement with the first reported findings on the role of KAT-Gcn5 and DUB Ubp8 into mitochondria that suggsts the combined use of epidrugs and inhibitors to traditional chemotherapy in order to achieve more effective results.

## Working Model

In conclusion, we propose a model from data collected in budding yeast. In the nucleus, an assembled SAGA complex exerts its canonical functions engaged in histone acetylation, opening of chromatin structure and activation of gene transcription. In addition to this *bona fide* nuclear activity, we propose a novel one with a role of KAT-Gcn5 and DUB-Ubp8 in yeast respiration. The mitochondrial localization of Gcn5 and Ubp8 as individual proteins supports their role not exclusively dependent on the assembly of the SAGA complex. Starting from these findings, we suggest to further study the human horthologs of Gcn5 and Ubp8 to understand correlations with mitochondria and their role in cancer cells metabolism.

## Author Contributions

All authors contributed to the writing and elaboration of this mini-review.

## Conflict of Interest Statement

The authors declare that the research was conducted in the absence of any commercial or financial relationships that could be construed as a potential conflict of interest.

## Footnotes

1 https://www.yeastgenome.org/
